# Aberrant Expression of Mitochondrial SAM Transporter SLC25A26 Impairs Oocyte Maturation and Early Development in Mice

**DOI:** 10.1155/2022/1681623

**Published:** 2022-04-13

**Authors:** Gui-ping Cheng, Shi-meng Guo, Ying Yin, Yuan-yuan Li, Ximiao He, Li-quan Zhou

**Affiliations:** ^1^Institute of Reproductive Health, Tongji Medical College, Huazhong University of Science and Technology, Wuhan, China; ^2^School of Basic Medicine, Tongji Medical College, Huazhong University of Science and Technology, Wuhan, Hubei, China

## Abstract

The immature germinal vesicle (GV) oocytes proceed through metaphase I (MI) division, extrude the first polar body, and become mature metaphase II (MII) oocytes for fertilization which is followed by preimplantation and postimplantation development until birth. *Slc25a26* is the gene encoding S-adenosylmethionine carrier (SAMC), a member of the mitochondrial carrier family. Its major function is to catalyze the uptake of S-adenosylmethionine (SAM) from cytosol into mitochondria, which is the only known mitochondrial SAM transporter. In the present study, we demonstrated that excessive SLC25A26 accumulation in mouse oocytes mimicked naturally aged oocytes and resulted in lower oocyte quality with decreased maturation rate and increased reactive oxygen species (ROS) by impairing mitochondrial function. Increased level of *Slc25a26* gene impacted gene expression in mouse oocytes such as *mt-Cytb* which regulates mitochondrial respiratory chain. Furthermore, increased level of *Slc25a26* gene in fertilized oocytes slightly compromised blastocyst formation, and *Slc25a26* knockout mice displayed embryonic lethality around 10.5 dpc. Taken together, our results showed that *Slc25a26* gene plays a critical role in oocyte maturation and early mouse development.

## 1. Introduction

In female mice, primordial germ cells (PGCs) initiate entry into prophase of meiosis I on embryonic day 13.5. Around the time of birth, oocytes are arrested at the diplotene stage in prophase I of meiosis for an extended period of time (up to months in mice and decades in humans) which is also called germinal vesicle (GV) stage [1, 2]. Upon hormonal surge, a limited number of immature oocytes resume meiosis which is indicated by GV breakdown. Accompanying with the chromatin condensation and microtubule organization, the oocytes proceed through the meiosis I (MI) division and extrude the first polar body (Pb1), and then, meiosis becomes arrested again at metaphase II (MII) until fertilization activates it to complete the second meiotic division [3–5]. Any errors during this process will affect meiotic progression and fertilization, producing low-quality oocytes [6]. In most mammals, oocyte quality declines with increase in maternal age [7]. Successful fertilization and subsequent embryonic development depend on oocyte quality which can be assessed by oocyte maturation, spindle formation, energy supply, syngamy, and early embryonic development and epigenetic modifications [8]. During oocyte maturation, mitochondria are the major source of energy supply of the oocytes, and changes of function and distribution of mitochondria may affect energy supply and oocyte maturation [8]. In addition, mitochondrial dysfunction also impairs early embryo development and can cause postimplantation failure [9].

SAM (S-adenosylmethionine) is the universal methyl donor involved in a broad range of biological methylation reactions [10, 11]. In the cytosol, SAM is synthesized from methionine and adenosine under the action of methionine adenosine transferase [12–14]. *Slc25a26* is the gene encoding S-adenosylmethionine carrier (SAMC), a member of the mitochondrial carrier family, which is the only known mitochondrial SAM transporter catalyzing the uptake of SAM from the cytosol into mitochondria [15]. In mitochondria, SAM is required for methylation of DNA, RNA, and proteins and as an intermediate in the biosynthesis of lipoic acid, ubiquinone [16]. Aberrant expression of SLC25A26 may induce various mitochondrial dysfunctions, leading to impairment of oxidative phosphorylation, increased oxidative stress, decrease in glutathione (GSH) defense and apoptosis, and impaired cellular functions by regulating methyl metabolism [15, 17]. In addition, impairment of SAMC function, as consequence of mutations in *Slc25a26*, causes various mitochondrial defects, including those affecting RNA stability, protein modification, mitochondrial translation, and the biosynthesis of CoQ10 and lipoic acid [18].

Thus, we asked whether *Slc25a26* plays an important role in determining oocyte quality by controlling mitochondrial functions. In present study, we used *Slc25a26*-overexpressed oocytes/embryos and knockout embryos to demonstrate that proper level of *Slc25a26* is important for oocyte maturation and early embryo development.

## 2. Materials and Methods

### 2.1. Animals

Young (4-5 weeks) and naturally aged ICR mice (10-12 months) were purchased from SIPEIFU (Beijing, China). *Slc25a26* knockout mice (C57 background) were obtained from Zhi-hua Wang Laboratory in the Wuhan University. All the mice were housed in SPF animal facility at the Huazhong University of Science and Technology.

### 2.2. Oocyte and Embryo Collection for Culturing and Microinjection

Fully grown GV oocytes were obtained from female mice by manual rupturing of antral ovarian follicles. For microinjection, denuded GV oocytes were collected in M2 medium (M1250, Easycheck, China) with 2.5 *μ*M milrinone to inhibit spontaneous germinal vesicle breakdown (GVBD). Then, *Slc25a26* cRNA (700 ng/*μ*l) were coinjected with *H2b-egfp* cRNA (200 *μ*g/*μ*l) into GV oocytes using a micromanipulator and microinjector (Eppendorf) under an inverted microscope (Eclipse TE200, Nikon). After microinjection, the oocytes were cultured in *in vitro* maturation (IVM) medium (M2115, Easycheck, China) with 2.5 *μ*M milrinone for 8 h to allow translation of exogenous cRNA. Similarly, siRNA against *Slc25a26* (70 *μ*M, sense: 5′-GCUGUUGGAUCCUUUCCUATT-3′; antisense: 5′-UAGGAAAGGAUCCAACAGCTT-3′) or negative control oligo was injected into GV oocytes and cultured in IVM medium with milrinone for 20 h to allow efficient knockdown. siRNA against mouse *Slc25a26* and the negative control oligo were synthesized by GenePharma (Shanghai, China). Then, microinjected oocytes were washed in IVM medium for several times, followed by culturing in IVM medium covered with mineral oil at 37°C in a 5% CO_2_ incubator for *in vitro* maturation. To collect zygotes, 3-4-week-old female mice were superovulated by injecting 10 IU of pregnant mare serum gonadotropin (PMSG) followed 48 h later with 10 IU of human chorionic gonadotropin (HCG) then mating with male overnight. Zygotes were obtained from the oviducts after 16 h of HCG injection. Zygotes were injected with *Slc25a26* cRNA together with *H2b-egfp* cRNA and cultured in KSOM medium (M1450, Easycheck, China) covered with mineral oil at 37°C in a 5% CO_2_ incubator for their development.

### 2.3. Plasmid Construction and cRNA Synthesis

Total RNA was extracted from mouse testis following the manufacturer's protocol using the TRI Reagent (Sigma, T9424). Total RNA was reverse transcribed into cDNA by Hifair 1st Strand cDNA Synthesis Super Mix for qPCR (gDNA digester plus) (11141ES10, YEASEN, Shanghai, China). *Slc25a26* gene was cloned from cDNA using nested PCR by Prime-STAR HS DNA Polymerase (R010A, TaKaRa) with 2 primer pairs (the first pair: forward, 5′-AAGTAGTTGCTCCATATCCCG-3′; reverse, 5′-TTGTAGCCAGTTTGCTTTCC-3′; the 2nd pair: forward, 5′-CTAGCTAGCCACCATGGACGCGCCGGGCT-3′; reverse, 5′-CTGCTAA CCGGTGGTGGGCTCTTCCTGCCCACC-3′). PCR products were digested with NheI and AgeI (NEB Inc., MA, USA) and ligated into PVAX1 vector with FLAG tag. cRNAs were *in vitro* transcribed and capped from linearized plasmids using mMESSAGE mMACHINE Kit (AM1344, Invitrogen) and Poly(A) Tailing Kit (AM1350, Invitrogen) according to the manufacturer's instruction. Synthesized RNA was aliquoted and stored at -80°C.

### 2.4. Immunofluorescence

Oocytes or embryos were fixed in 4% paraformaldehyde for 30 min and permeabilized in 0.5% Triton X-100 for 25 min at room temperature (RT). After blocking with 1% BSA for 1 h at RT, oocytes were incubated with primary antibodies at 4°C overnight. Primary antibodies used were as follows: anti-*α*-tubulin-FITC antibody (1 : 200; Sigma, F2168), anti-SLC25A26 antibody (1 : 150; Sigma, HPA026887), and anti-FLAG antibody (1; 200; AE063, ABclonal). Then, oocytes were washed in PBS 3 times and incubated with CoraLite594-conjugated Goat Anti-Rabbit IgG(H+L) (SA00013-4, Proteintech Group, Inc.) and CoraLite488-conjugated Affinipure Goat Anti-Rabbit IgG(H+L) (SA00013-2, Proteintech Group, Inc.) for 1-2 h at RT. After washing in PBS, oocytes were put into the antifade medium containing Hoechst 33342 on a glass slide and observed under a confocal laser scanning microscope (LSM 800, Zeiss, Germany). For mitochondria and endoplasmic reticulum staining, oocytes were cultured in M2 medium containing 200 nM Mito-Tracker Red CMXRos (C1035, Beyotime, China) or ER-Tracker Red (C1041, Beyotime, China) with a dilution ratio of 1 : 1000 for 30 min at 37°C. To assess mitochondrial membrane potential, oocytes were cultured in M2 medium with JC-1 (C2006, Beyotime, China) at 37°C for 30 min. After three times of washing, oocytes were mounted on antifade medium with Hoechst 33342 and examined under a confocal laser scanning microscope (LSM 800, Zeiss, Germany).

Reactive oxygen species (ROS) levels of oocytes were determined by Reactive Oxygen Species Assay Kit (S0033S, Beyotime, China). Generally, oocytes were incubated with fluorescent probe DCFHDA for 30 min at 37°C. Following washing, oocytes were placed into antifade medium containing Hoechst 33342 and observed under the laser scanning confocal microscope (LSM 800, Zeiss, Germany). For Annexin-V staining, oocytes were stained with the Annexin V-FITC (C1062L, Beyotime, China) for 20 min at RT in the dark. Then oocytes were washed three times and placed into antifade medium containing Hoechst 33342 and observed under the laser scanning confocal microscope (LSM 800, Zeiss, Germany). Immunofluorescence was carried out and processed on control oocytes and treatment in parallel for each antibody. The same confocal microscope settings were used to obtained images for comparison. Immunofluorescence signals were captured using Image J 1.43u software (National Institutes of Health (NIH)), and mean gray value of regions of interest was analyzed for the quantification of the fluorescent intensity.

### 2.5. Western Blotting Analysis

GV oocytes were lysed in 1 × SDS-PAGE Protein Loading Buffer (5 × SDS-PAGE Protein Loading Buffer (20315ES05, YEASEN, China) diluted with RIPA lysis buffer containing protease inhibitor) and boiled in 98°C water for 5 minutes. The lysates were then separated by 12% sodium dodecyl sulfate-polyacrylamide gel electrophoresis and transferred onto polyvinylidene difluoride membranes (Millipore, Darmstadt, Germany). Following blocking in 5% nonfat milk for 1 h at room temperature, the blots were incubated with anti-SLC25A26 (1 : 500 dilution, Sigma, HPA026887) and anti-GAPDH (1 : 1000 dilution, 60004-1-Ig, Proteintech, Wuhan, China) antibodies overnight at 4°C. After washing in TBST for 3 times, the blots were incubated with horseradish peroxidase-conjugated secondary antibodies for 1 h at room temperature. After washing in TBST, chemiluminescence was detected with ECL working fluid (Beyotime). The relative densities of protein bands were analyzed using ImageJ software.

### 2.6. Measurement of ATP Content

ATP determination was performed using an ATP Determination Kit (A22066, Invitrogen). In brief, 15 oocytes were lysed in 30 *μ*l lysis buffer (20 mM Tris, 0.9% Nonidet P-40, and 0.9% Tween-20). Low-concentration ATP standard solutions (0, 0.1, 0.5, 1, 10, 50, 100, and 500 nmol of ATP) were prepared by diluting the 5 mM ATP solution. 10 *μ*l samples or ATP standard solutions mixed with 200 *μ*l standard reaction solution (1× reaction buffer, 1 mM DTT, 0.5 mM D-luciferin, and 0.2 *μ*g/ml firefly luciferase) were made according to the experimental protocol of ATP Determination Kit, and bioluminescence was measured using a plate reader. An eight-point standard curve was produced in each assay, and ATP levels were calculated using the formula derived from the linear regression of the standard curve.

### 2.7. Determination of mtDNA Copy Number

10-20 oocytes were loaded in a PCR tube with 30 *μ*l lysis buffer (50 mM Tris-HCl pH 8.0,;0.5% Triton X-100, and 0.2 mg/ml proteinase K) and was incubated at 55°C for 2 h. Proteinase K was heat inactivated at 95°C for 10 min, and then, the samples were used for PCR analysis. Quantitative real-time PCR was performed using the ABI StepOne system and mouse mtDNA-specific primers are as follows: forward, 5′-AACCTGGCACTGAGTCACCA-3′ and, reverse, 5′-GGGTCTGAGTGTATATATCATGAAGAGAAT-3′.

### 2.8. Low-Input RNA Sequencing and Data Analysis

RNA sequencing library preparation of oocytes was carried out by the Smart-Seq2 method. Generally, samples (10 oocytes per sample) for the control and SLC25A26-OE groups were collected and directly lysed in the single-cell lysis components with RNase inhibitors. Then, the 1st cDNA strand was synthesized with oligo dT by reverse transcription and amplified by PCR. Following purification of amplified products, the library was constructed by DNA fragmentation, end repair, addition of “A” and joint, PCR amplification, and library quality control. Qualified libraries were loaded onto Illumina Hiseq platform for PE150 sequencing. Raw reads were processed with Cutadapt v1.16. Trimmed reads were mapped to mouse genome (GENCODE release M23) using STAR with default settings. Differential expression of genes for pairwise comparisons was assessed by DESeq2 v1.24.0. The TE transcripts program was used to obtain counts for transposable elements (TEs) with default parameters. Read counts of gene and TE transcripts were normalized to the total aligned counts. Differentially regulated genes in the DESeq2 analysis were defined as those which were more than twofold increased or decreased with adjusted *p* < 0.05. Volcano and heat map were generated by R.

### 2.9. RNA Extraction and Quantitative Real-Time PCR (qRT-PCR)

A total of 25-30 oocytes/zygotes or 20 cleavage embryos or 15 blastocysts were collected, respectively. Total RNA was extracted using the TRI Reagent (Sigma, T9424) and reverse transcribed into cDNA by Hifair 1st Strand cDNA Synthesis Super Mix for qPCR (gDNA digester plus) (11141ES10, YEASEN, Shanghai, China). Each PCR reaction consisted of 10 *μ*l of Hieff qPCR SYBR Green Master Mix (High Rox Plus) (11203ES03, YEASEN, Shanghai, China), 8.2 *μ*l of water, 1 *μ*l of cDNA sample, and 0.8 *μ*l of gene-specific primers (5 *μ*M; *Slc25a26* primers: forward, 5′-TCTGGGGCAACAGTGTGTAG-3′; reverse, 5′-TACTAAGTGTGTGCGGCGGT-3′; *mt-Cytb* primers: forward, 5′-ACCTCAAAGCAACGAAGCCT-3′; reverse, 5′-TGGGTGTTCTACTGGTTGGC-3′). Real-time PCR was performed by the StepOnePlus Real-Time PCR system (Applied Biosystems, USA). mRNA levels were normalized to *Gapdh*. The experiments were performed for at least three times.

### 2.10. Genotyping

Total genomic DNA extracted from mouse tails was used to determine the genotypes of mice by PCR. Mouse tails were dissolved in a DNA lysis buffer (25 mM NaOH; 2 mM EDTA) at 95°C for 1 h and then in neutralizing buffer (40 mM Tris-HCl, pH = 6.8). PCR was performed for 30-33 cycles at 95°C for 30 s, 60°C for 30 s, and 72°C for 40 s, with a final extension at 72°C for 5 min using primers (knockout allele: forward, 5′-CCAGTGGATTGAAGAAGTTTTGAAGGAC-3′; reverse, 5′-AAGCTCCTTGTTGACTGGGTCATTC-3′; wild-type allele: forward, 5′-GAGGTAGAAGTGCAACTGAGCGAACA-3′, reverse, 5′-GTATCTGTACTGTTCCTGTGCATGGG-3′). DNA fragments were visualized by 2% agarose gel electrophoresis.

### 2.11. Analysis of Dissected Embryos

For the analysis postimplantation embryos, *Slc25a26* heterozygous males were mated with *Slc25a26* heterozygous females, and embryos were dissected from pregnant females at 7.5, 8.0, 8.5, 9.5, and 10.5 dpc (days post coitum). The day of plug formation was defined as embryonic day 0.5. After dissection, embryos were photographed with stereomicroscope linked to a camera.

### 2.12. Statistical Analysis

Data were presented as mean ± SEM. All experiments were replicated more than three times. Statistical comparisons were made with Student's *t* test. *p* <0.05 was considered to be statistically significant.

## 3. Results

### 3.1. Changes of mRNA and Protein Levels of *Slc25a26* during Mouse Oocyte Maturation and Preimplantation Development

**Figure 1:**
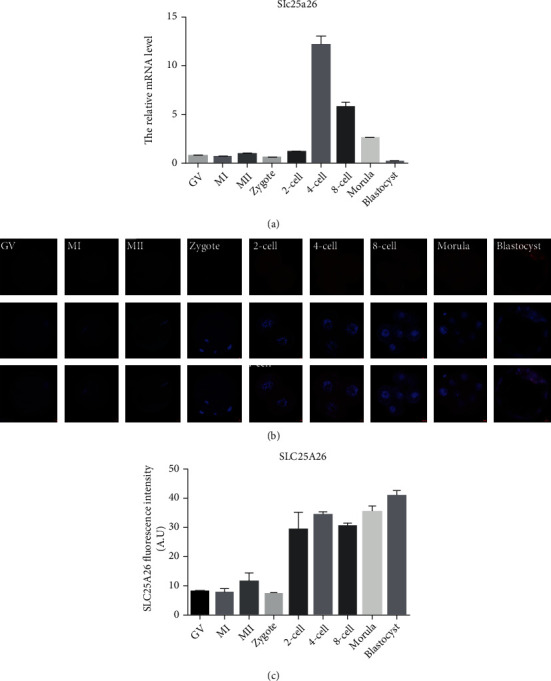
Expression pattern of mRNA and protein of *Slc25a26* during mouse oocyte maturation and embryo preimplantation development. (a) The mRNA levels of *Slc25a26* in GV, MI, and MII oocytes and mouse embryos at zygote, 2-cell, 4-cell, 8-cell, morula, and blastocyst stages were examined by qRT-PCR. 20–30 oocytes and embryos were collected for each group. (b) GV, MI, and MII oocytes and zygote, 2-cell, 4-cell, 8-cell, morula, and blastocyst stage embryos were immunolabeled with anti-SLC25A26 antibody to identify subcellular localization and expression of SLC25A26 during oocyte maturation and preimplantation development. Red, SLC25A26; blue, DNA. Bar = 10 *μ*m. (c) The protein levels of SLC25A26 in GV, MI, and MII oocytes and embryos at zygote, 2-cell, 4-cell, 8-cell, morula, and blastocyst stages were measured with ImageJ for quantification of fluorescence intensities (*n* = 8 for each group). A.U. means arbitrary units. Data are presented as the mean ± SEM.

The mRNA and protein levels of *Slc25a26* during oocyte maturation and preimplantation development were determined by qRT-PCR and immunofluorescence staining, respectively. The transcript of *Slc25a26* was at a low level in oocytes and early embryos before 4-cell stage, increased significantly at the 4-cell stage, then decreased gradually, and became weakest at blastocyst stage (Figure 1(a)). Immunofluorescence staining proved that SLC25A26 protein was mainly localized in the cytoplasm of oocytes and preimplantation embryos (Figure 1(b)). The fluorescence intensity of SLC25A26 appeared weak in oocytes and zygotes, was enhanced at the 2-cell stage, and had robust expression later on (Figures 1(b) and 1(c)). These results indicate that oocytes maintain a low level of SLC25A26 and SLC25A26 is induced after embryonic program is turned on.

### 3.2. Increased SLC25A26 Level in Naturally Aged Oocytes Impaired Developmental Competence of Mouse Oocytes

**Figure 2:**
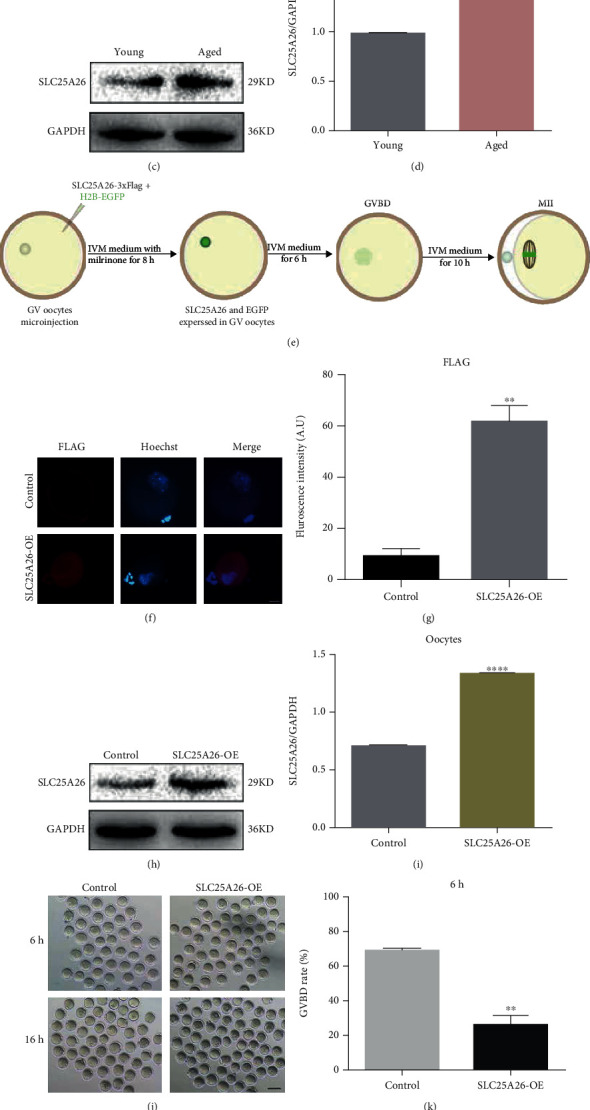
Effects of SLC25A26 expression on the meiotic progression of mouse oocytes. (a) Representative images of SLC25A26 protein staining in young and aged oocytes. Scale bar = 20 *μ*m. (b) The fluorescence intensity of SLC25A26 signals was measured in young and aged oocytes. ^∗∗∗^*p* < 0.001. A.U. means arbitrary units. (c) Representative western blotting images of SLC25A26 protein in young and aged oocytes. (d) The mean densitometric quantification of SLC25A26 protein level as measured in young and aged oocytes with ImageJ. ^∗∗∗∗^*p* < 0.0001. (e) Schematic of overexpressing the SLC25A26 in GV oocyte by microinjection and its IVM culture. Efficiency of SLC25A26 overexpression (SLC25A26-OE) after mRNA injection was verified by immunofluorescence: (f) representative images of FLAG protein staining in control and SLC25A26-OE group. Scale bar = 50 *μ*m. (g) The fluorescence intensity of FLAG signals was measured in control and SLC25A26-OE oocytes. ^∗∗^*p* < 0.01. A.U. means arbitrary units. (h) SLC25A26 protein level was determined by western blot analysis. (i) The mean densitometric quantification of SLC25A26 protein level as measured in control and SLC25A26-OE oocytes with ImageJ. ^∗∗∗∗^*p* < 0.0001. (j) Representative images of control and SLC25A26-OE oocytes. Scale bar = 100 *μ*m. (k and l) Quantitative analysis of GVBD and Pb1 extrusion rate in control and SLC25A26-OE. ^∗∗^*p* < 0.01 and ^∗∗∗∗^*p* < 0.0001. Data are presented as the mean ± SEM.

Firstly, we detected the SLC25A26 level in young and aged oocytes by immunostaining and western blotting. We identified significantly increased expression level in aged oocytes comparing to young oocytes (Figures 2(a), 2(b), 2(c), and 2(d)). To investigate the consequence of SLC25A26 protein accumulation in oocytes, we overexpressed SLC25A26 protein in GV oocytes by microinjection with *Slc25a26* cRNA, and *H2b-egfp* cRNA was coinjected to confirm success of microinjection (Fig. S1). The injected oocytes were then cultured in IVM medium containing milrinone to allow protein synthesis for 8 h and were then transferred to IVM medium for oocyte maturation (Figure 2(e)). The expression of exogenous SLC25A26 protein was verified by examination of FLAG tag and total SLC25A26 level (Figures 2(f), 2(g), 2(h), and 2(i)). After culturing for 6 hours in IVM medium, the ratio of GVBD was decreased in the SLC25A26 overexpression group compared with that in the control group (*p* < 0.01; Figures 2(j) and 2(k)). Moreover, the first polar body (Pb1) extrusion rate had significantly decline (*p* < 0.0001) due to SLC25A26 overexpression upon *in vitro* maturation for 16 hours (Figured 2(j) and 2(l)). These observations imply that SLC25A26 accumulation in mouse oocytes dampened developmental competence of oocytes.

To explore whether knockdown of *Slc25a26* could restore the quality of the aged oocytes, we injected siRNA against *Slc25a26* in aged GV oocytes (Fig. S2A). qRT-PCR was performed to validate decreased of *Slc25a26* transcript (Fig. S2B). However, we observed that there was no significant difference of GVBD rate and Pb1 extrusion rate between the aged oocytes and aged oocytes with *Slc25a26* knockdown (Fig. S2A, C). There are other misregulated factors that contribute to the decline of quality of aged oocytes, and knockdown of SLC25A26 is not enough to efficiently rescue maturation rate of aged oocytes.

### 3.3. Increased SLC25A26 Protein Level Damaged Spindle Organization and Chromosome Alignment in Mouse Oocytes

**Figure 3:**
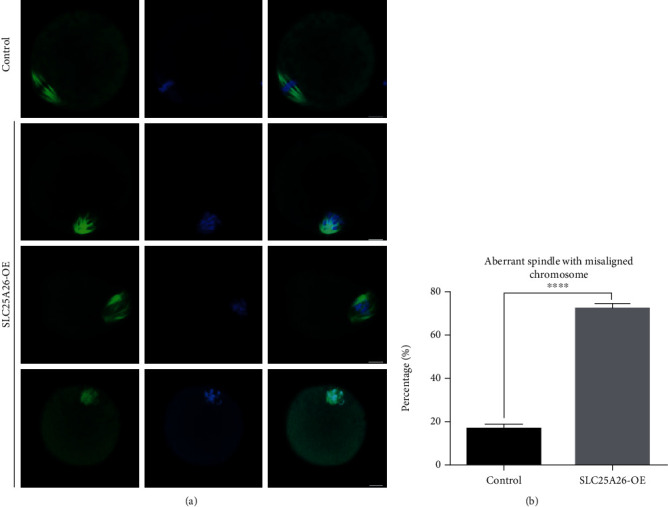
SLC25A26 overexpression induces spindle defects and chromosome misalignment in oocyte meiosis. (a) Control and SLC25A26-OE oocytes were stained with anti-*α*-tubulin-FITC antibody to visualize the spindle (green) and counterstained with Hoechst 33342 (blue) to observe chromosomes. Scale bar = 50 *μ*m. (b) The rate of control and SLC25A26-OE oocytes with spindle/chromosome defects was recorded. Data are presented as the mean ± SEM. ^∗∗∗∗^*p* < 0.0001.

One of the important indicators of high-quality oocytes is normal spindle morphology with aligned chromosomes which is associated with efficient oocyte meiosis [19]. Impaired oocyte maturation indicated that SLC25A26 overexpression affected meiotic apparatus in oocytes. Therefore, we stained the oocytes with anti-*α*-tubulin-FITC antibody to visualize the spindle morphologies and costained with Hoechst 33342 to detect chromosome alignment. The immunofluorescence results showed that control metaphase-stage oocytes displayed a typical barrel-shaped spindle with well-organized chromosomes at the equator plate (Figure 3(a)). By contrast, a high percentage of disorganized spindle morphologies and misaligned chromosomes was observed in SLC25A26-overexpressed oocytes (Figures 3(a) and 3(b); *p* < 0.0001). We observed irregular shaped spindles, some with one spindle pole and clumped chromosomes in oocytes with elevated SLC25A26 level. Therefore, SLC25A26 accumulation impaired spindle formation and chromosome arrangement in mouse oocytes.

### 3.4. Elevated SLC25A26 Level Caused Mitochondrial Dysfunction in Mouse Oocytes

**Figure 4:**
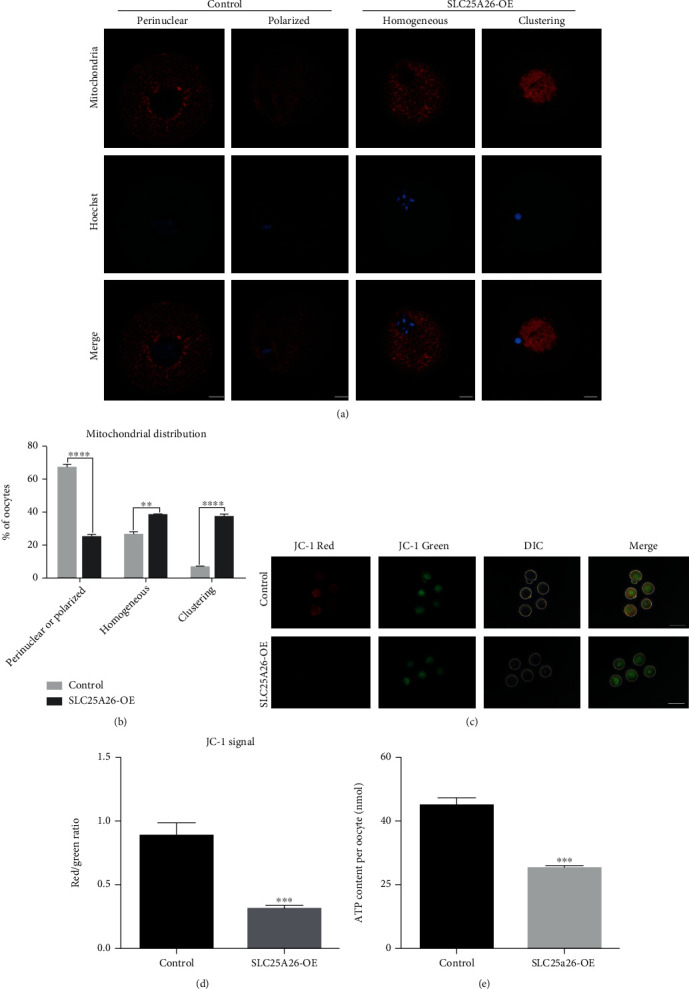
SLC25A26 overexpression disrupts mitochondrial redistribution, membrane potential, and ATP content in oocyte. (a) Representative images of mitochondrial distribution in control and SLC25A26-OE oocytes. Oocytes were stained with Mito-Tracker Red to show mitochondria. Scale bar = 10 *μ*m. (b) Each mitochondrial distribution pattern rate was recorded in in control and SLC25A26-OE oocytes. ^∗∗^*p* < 0.01 and ^∗∗∗∗^*p* < 0.0001. (c) Mitochondrial membrane potential (ΔΨm) was detected by JC-1 staining in control and SLC25A26-OE oocytes (red, high *ΔΨ*m; green, low *ΔΨ*m). Scale bar = 100 *μ*m. (d) The ratio of red to green fluorescence intensity was calculated in control and SLC25A26-OE oocytes. (e) ATP levels were measured in control and SLC25A26-OE oocytes. Data are presented as the mean ± SEM. ^∗∗∗^*p* < 0.001.

Due to SLC25A26's function in mitochondria, we then checked whether mitochondrial functions were affected upon *Slc25a26* overexpression. Because mitochondrial morphology and dynamics are tightly related to mitochondrial function, we labeled oocyte mitochondria with Mito-Tracker to visualize their distribution patterns. Normally, the majority of immature oocytes presented perinuclear distribution pattern with accumulation of mitochondria around nucleus, and MII oocytes displayed a polarized distribution pattern with mitochondria enriched around the spindle [20]. We found that the proportion of clustering and homogeneous mitochondria in oocytes were significantly increased when SLC25A26 was overexpressed (Figures 4(a) and 4(b); *p* < 0.0001 and *p* < 0.01), whereas the proportion of perinuclear/polarized distribution pattern was declined accordingly (Figures 4(a) and 4(b); *p* < 0.0001). We also detected mtDNA copy number in SLC25A26-overexpressed and control oocytes. However, there was no significant difference between the two groups (Fig. S3). The key function of the mitochondria is to produce ATP. Therefore, the ATP level is an ingenious indicator of mitochondrial function. We then measured the ATP content in oocytes and identified significantly reduced ATP content of SLC25A26-overexpressed oocytes compared with control oocytes (Figure 4(e); *p* < 0.001). Additionally, mitochondrial membrane potential (ΔΨ*m*) is critical to ATP production by ATP synthase and a measure of mitochondria viability [21]. We then assessed mitochondrial membrane potential by JC-1 staining. Mitochondria with high membrane potential presented red fluorescence and low membrane potential exhibited green fluorescence. For quantitative analysis, the ratios of red/green fluorescence intensity were calculated to present Δ*ψm*. Notably, the red/green ratio was significantly decreased in SLC25A26-overexpressed oocytes as compared to those of control (Figures 4(c) and 4(d); *p* < 0.001). These results showed that SLC25A26 accumulation in oocytes had a general disturbance of mitochondrial functions.

### 3.5. Accumulation of SLC25A26 Induced Oxidative Stress and Apoptosis in Mouse Oocytes

**Figure 5:**
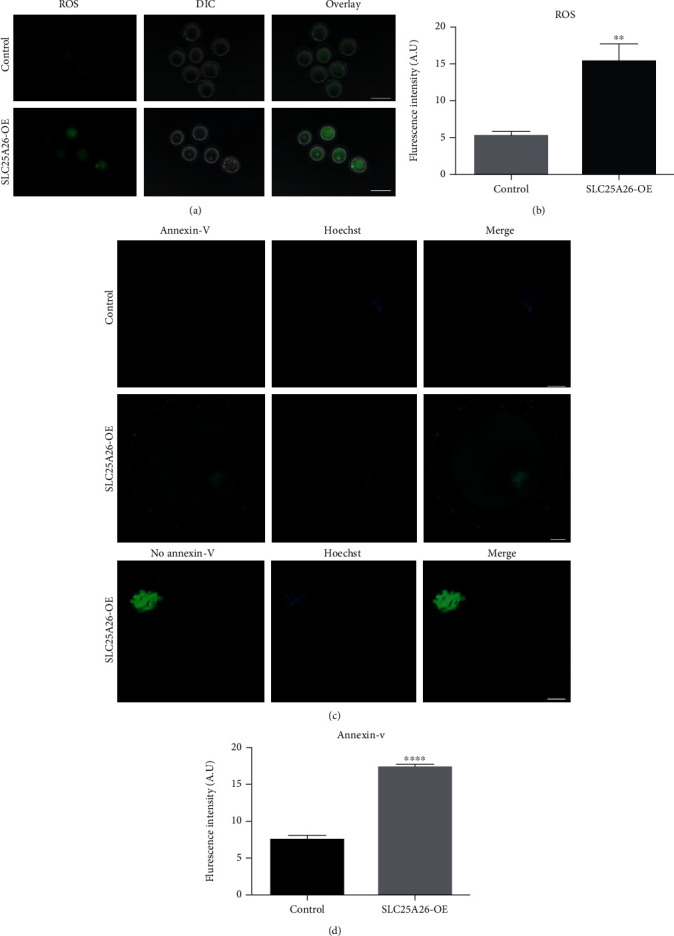
Effect of SLC25A26 overexpression on ROS accumulation and apoptosis in oocytes. (a) Representative images of ROS levels detected by DCFH staining in control and SLC25A26-OE oocytes. Scale bar = 100 *μ*m. (b) The fluorescence intensity of ROS signals was measured in control and SLC25A26-OE oocytes. (c) Representative images of apoptotic status, assessed by Annexin-V staining, in control and SLC25A26-OE oocytes. Scale bar = 10 *μ*m. (d) The fluorescence intensity of Annexin-V signals was measured in control and SLC25A26-OE oocytes. A.U. means arbitrary units. Data in (b) and (d) are presented as mean ± SEM. ^∗∗^*p* < 0.01 and ^∗∗∗∗^*p* < 0.0001.

Mitochondria are the main producer and target of reactive oxygen species (ROS) [22, 23]. When mitochondria are injured in oocyte, oxidative stress response may be activated and promote apoptosis. Therefore, we detected ROS levels using DCFH-DA fluorescent dye staining and apoptosis by Annexin-V staining both in control and SLC25A26-overexpressed oocytes. As shown in Figures 5(a) and 5(b), the fluorescence intensity of ROS in SLC25A26-overexpressed oocytes was significantly increased compared to control (*p* < 0.01). As anticipated, Annexin-V staining showed no fluorescence signal in control oocytes. However, positive fluorescence signals were detected in SLC25A26-overexpressed oocytes (Figures 5(c) and 5(d); *p* < 0.0001), indicating the occurrence of early apoptosis. Our results showed that enhanced SLC25A26 activity induced oxidative stress and early apoptosis in mouse oocytes.

### 3.6. Effects of SLC25A26 Overexpression on Mouse Oocyte Transcriptome

**Figure 6:**
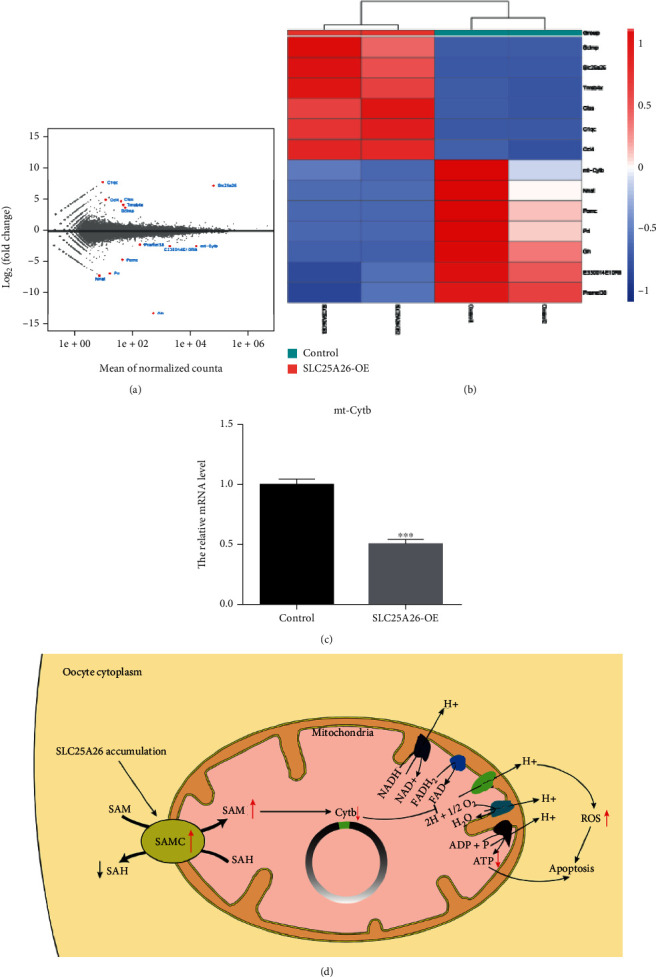
Transcriptome comparison between control and SLC25A26 overexpression oocytes. (a) An MA plot showing the RNA expression level. The changed genes were designated by red (up- or downregulated) in SLC25A26-OE relative to control oocytes. (b) Heatmap illustration displaying the 13 changed genes after SLC25A26-OE oocytes. (c) The relative mRNA level of *mt-Cytb* were examined in control and SLC25A26-OE oocytes by qRT-PCR. ^∗∗∗^*p* < 0.001. Data are presented as the mean ± SEM. (d) Schematic pathways related to SLC25A26 overexpression. SLC25A26 (SAMC) overexpression leads to an increase of SAM level, which affects the synthesis of mt-Cytb (complexes III) of the respiratory chain and leads to insufficient synthesis of ATP. Downregulated mt-Cytb also caused elevated ROS.

To further explore the mechanisms for declined ability of oocyte maturation caused by SLC25A26, we performed ultra low-input RNA-seq analysis to characterize change of transcriptome of SLC25A26-overexpressed oocytes. Principal component analysis (PCA) showed variation of transcriptome between control and SLC25A26-overexpressed GV oocytes (Fig. S4). Notably, only a few genes had changed expression upon SLC25A26 overexpression, with 6 genes upregulated and 7 genes downregulated (Figures 6(a) and 6(b)). *Slc25a26* is one of the 6 upregulated genes, which is consistent with the overexpression purpose of our experiment. *C1qc*, *Ccl4*, *Ctss*, *Tmsb4x*, and *Scimp* were other 5 upregulated genes and *Pramel38*, *mt-Cytb*, *E330014E10Rik*, *Pomc*, *Prl*, *Nnat*, and *Gh* were 7 downregulated genes.  Table 1 annotates the 12 genes with changed expression. Four upregulated genes (*C1qc*, *Ccl4*, *Ctss*, and *Scimp*) are related to immunity, and *Tmsb4x* function is associated with binding to and sequestering actin monomers (G actin) to inhibit actin polymerization. Among 7 downregulated genes, 3 genes (*Pomc*, *Prl*, and *Gh*) belong to hormones and 2 genes (*Pramel38* and *E330014E10Rik*) are associated with apoptotic process, cell differentiation, and transcription. Interestingly, *mt-Cytb* is a component of the ubiquinol-cytochrome c reductase complex (complex III or cytochrome b-c1 complex) that is a part of the mitochondrial respiratory chain. In addition, qRT-PCR was performed to validate decreased expression of *mt-Cytb* (Figure 6(c)).

Collectively, RNA-seq result indicated that SLC25A26 overexpression altered transcriptome of oocytes which may reflect impaired oocyte quality. We also analyzed the level of TEs and observed no obvious changes upon SLC25A26 overexpression (Fig. S5).

### 3.7. Aberrant Expression of SLC25A26 Disturbed Early Mouse Development

**Figure 7:**
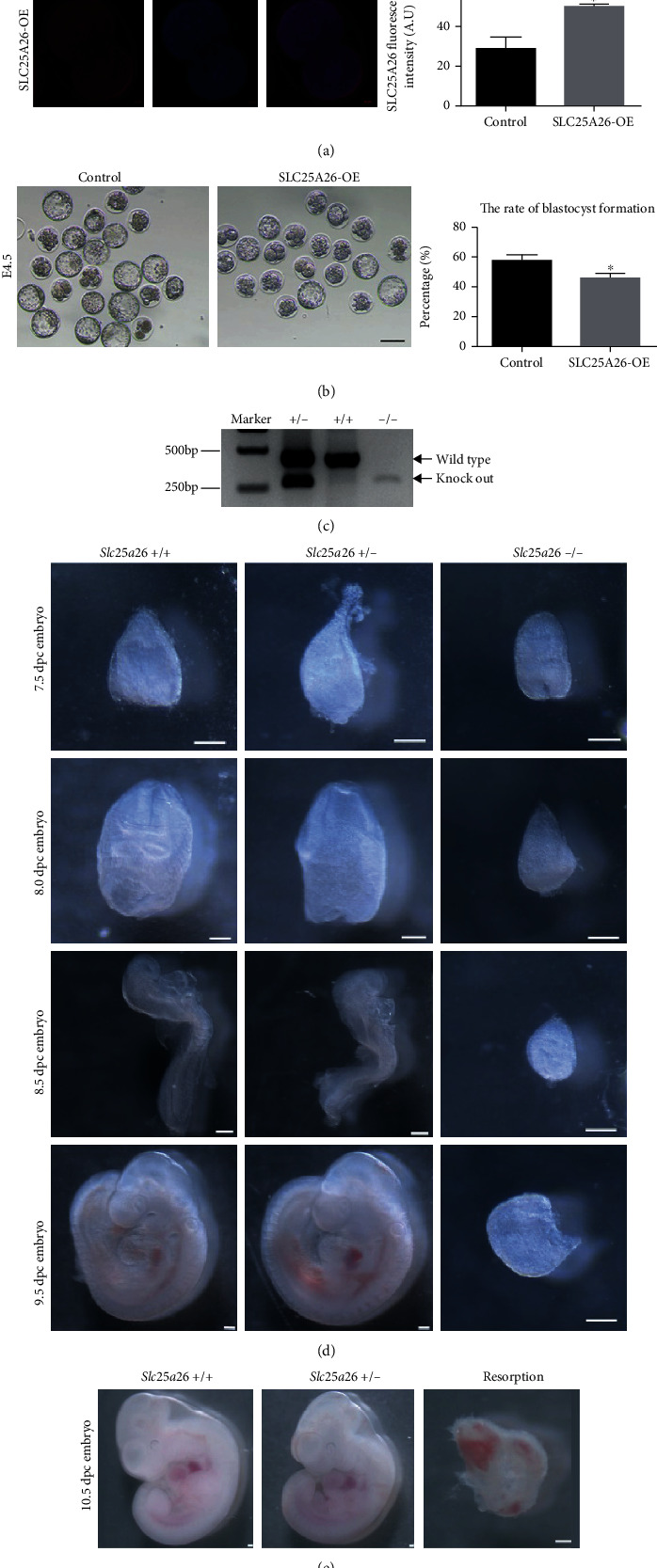
Effects of SLC25A26 overexpression on preimplantation embryos and SLC25A26 knockout on postimplantation embryo development. (a) Representative images of SLC25A26 protein staining in control and SLC25A26-OE 2-cell embryos. Scale bar = 10 *μ*m. The fluorescence intensity of SLC25A26 signals was measured in control and SLC25A26-OE 2-cell embryos with ImageJ. A.U. means arbitrary units. Data are presented as the mean ± SEM. ^∗^*p* < 0.05. (b) Representative image of E4.5 control and SLC25A26-OE embryos. Scale bar = 100 *μ*m. And the blastocyst formation rate was recorded in control and SLC25A26-OE embryo after fertilization. Data are presented as the mean ± SEM. ^∗^*p* < 0.05. (c) Representative image of genotyping PCR products visualized on agarose gel. The bands indicated by arrow were amplified from wild-type and knockout allele, respectively. (d) Representative image of embryos at 7.5, 8.0, 8.5, and 9.5 dpc obtained by crossing *Slc25a26* heterozygous mice. Wild-type (+/+), heterozygous (+/–), and knockout embryos are shown. Scale bar = 100 *μ*m. (e) Representative image of embryos at 10.5 dpc. Wild-type (+/+), heterozygous (+/–), and resorption embryos are shown. Scale bar = 100 *μ*m.

To evaluate the impact of SLC25A26 accumulation in preimplantation development, we overexpressed SLC25A26 in mouse zygotes by microinjecting *Slc25a26* cRNA. And H2b-egfp cRNA was coinjected to confirm success of microinjection (Fig. S6). Following microinjection, the expression of exogenous SLC25A26 protein was verified by immunostaining examination of SLC25A26 in 2-cell embryos (Figure 7(a)). The rate of blastocyst formation was evaluated at E4.5. Compared with the control group, the blastocyst formation rate of the SLC25A26 overexpression group was slightly decreased (Figure 7(b); *p* < 0.05). This result revealed that SLC25A26 accumulation had minor effect on preimplantation development.

To further identify developmental roles of *Slc25a26*, we examined phenotype of *Slc25a26* knockout mice. In the knockout mouse model, exon 2 of *Slc25a26* was deleted with Clustered Regularly Interspaced Short Palindromic Repeats/CRISPR-associated protein 9 (CRISPR/Cas9) technology (Fig. S7). Genotyping PCR products are shown in Figure 7(c) and strategy of genotyping is shown in Fig. S8. To obtain homozygous knockout mice, we mated heterozygous male mice with heterozygous female mice and noticed that although most obtained early embryos could develop to blastocyst stage, no homozygous pups were obtained, indicating developmental arrest at postimplantation stage. Therefore, we isolated embryos at 7.5, 8.0, 8.5, 9.5, and 10.5 dpc for phenotype examination. No obvious morphological changes could be seen in heterozygous embryos as compared to the wild type (Figures 6(d) and 6(e)), and *Slc25a26* knockout embryos (*Slc25a26*-/-) were also identified in 7.5, 8.0, 8.5, and 9.5 dpc embryos (Figure 7(d)). Notably, the 7.5 dpc embryos were seen as morphologically comparable to heterozygous or wild-type embryos. However, 8.0, 8.5, and 9.5 dpc embryos of *Slc25a26*-/- embryos had obvious developmental arrest compared with the wild-type control embryos. We also observed resorbed embryos in 10.5 dpc embryos (Figure 7(e)) where the genotype could not be precisely identified since the mother's decidual tissue could not be separated from the resorptions. No *Slc25a26*-/- embryos were found at these or further stages. The above results clearly indicated that *Slc25a26* deficiency sabotaged early mouse development.

## 4. Discussion

Mitochondrion is the major place for aerobic respiration which is the primary source of ATP through oxidative phosphorylation (OXPHOS). Beyond the production of ATP via OXPHOS, mitochondria participate in numerous cellular functions, involved in stress responses, apoptosis, and chromosome segregation [9]. Disturbances and deficiencies of mitochondrial function not only reduce quality of oocyte/embryo but also contribute to postimplantation failure, long-term cell dysfunction, and adult disease [9, 24]. According to mRNA and protein level of *Slc25a26* in our study, it seems that SLC25A26 is dispensable for development of oocytes and early stage of embryos. Interestingly, we found that SLC25A26 level was increased in oocytes from naturally aged mice which implied that enhanced expression of SLC25A26 might impact oocyte quality. Indeed, our examination showed that SLC25A26-overexpressed oocytes exhibited lower GVBD and maturation rate. As expected, SLC25A26 overexpression in oocytes led to aberrant mitochondrial distribution, reduced Δ*ψm* and ATP content, and increased production of ROS. All of these mitochondrial abnormalities can impair oocyte quality. For example, the difference in mitochondrial localization has been associated with developmental competence of oocytes [25]. Moreover, mitochondrial dysfunction has been associated with overproduction of ROS which leads to oxidative stress and reduced ATP [26–28].

SLC25A26 is a member of mitochondrial carrier family, and its main function is to catalyze the import of SAM into mitochondria in exchange for intramitochondrial S-adenosine homocysteine (SAH) [16, 29, 30]. SAH was produced from SAM in the methylation reactions and only hydrolyzed in the cytoplasm [16, 29, 30]. The overexpression of SLC25A26 upregulated SAM entry into mitochondria forcing methionine to be converted to SAM. Thus, homocysteine was accumulated and GSH was depleted [15]. GSH is a detoxifying antioxidant that protects the cells from ROS as well as free radicals [31]; homocysteine is a toxic metabolite to tissues in high concentrations associated with abnormal gene expression and induced cell cycle arrest and cell apoptosis [15, 32–34]. Hyperhomocysteinemia (HHcy) impairs oocyte/embryo quality via deregulation of one-carbon metabolism and hypermethylation of mitochondrial DNA [35, 36]. The overexpression of SLC25A26 may be similar to SAMC overexpression in CaSki cells inducing abnormal homocysteine and GSH level which triggered ROS production and apoptosis [15].

Furthermore, through ultra low-input RNA-seq analysis, we observed a marked reduction of mtDNA-encoded cytochrome b, *mt-Cytb*, in SLC25A26-overexpressed oocytes. *mt-Cytb* is the only mtDNA-encoded subunit of the ubiquinol-cytochrome c reductase complex (complex III or cytochrome b-c1 complex) that is part of the mitochondrial respiratory chain [37, 38]. Mitochondrial respiratory chain (also called mitochondrial electron transport chain (ETC)), composed of four multiprotein complexes named complex I–IV, is a series of electronic carriers embedded in the mitochondrial inner membrane to form proton gradient used for the generation of ATP [38, 39]. Besides its involvement in ATP synthesis, the mitochondrial ETC promotes ROS production [40]. ETC I and III complexes are the major sources of ROS [41]. Many studies demonstrated that downregulated *mt-Cytb* caused elevated ROS and decreased ATP production [37, 38, 42, 43]. Oocyte meiotic maturation is an active energy demanding process, and decreased ATP content in oocytes were frequently found to be related to defective spindle assembly and chromosome alignment [44, 45]. Thus, impaired oocyte quality upon SLC25A26 overexpression may be due to disturbed DNA methylation in mitochondrial genome for decreased expression of *mt-Cytb*. Moreover, we observed altered expression of a few nuclear genes upon SLC25A26 overexpression, and this may be caused by indirectly impacted degradation activity of transcripts in oocytes, possibly through interrupted mitochondrial activity.

Additionally, we found that *Slc25a26* knockout mice displayed embryonic lethality at 10.5dpc, indicating an essential role of SLC25A26 for early embryo development. Accumulated evidence also showed that SLC25A26 plays an important role in human development. Clinical findings reported that SLC25A26 mutations were associated with a complex autosomal recessive multisystem disease COXPD28. Patients with SLC25A26 mutations ranged from neonatal mortality resulting from respiratory insufficiency and hydrops to childhood acute episodes of cardiopulmonary failure and slowly progressive muscle weakness [18, 46]. These articles elucidated that SLC25A26 mutations caused various mitochondrial defects, including those affecting RNA stability, protein modification, mitochondrial translation, and the biosynthesis of CoQ10 and lipoic acid [18, 46]. Knockout of *Slc25a26* in mice caused embryo-lethal phenotype which may be due to failure of the mitochondrial biogenesis during embryogenesis. The critical cellular roles of mitochondria are underscored by the observation that loss of mitochondrial fusion/fission results in embryonic lethality in mice [9, 47]. Mice deficient in either *Mfn1* or *Mfn2* interrelated mitochondrial fusion die around 11.5 dpc [48]. Another mitochondrial fusion-related factor *Opa1* knockout in mice caused embryonic lethality at 13.5dpc [49]. Knockout of dynamin-related protein 1 (*Drp1*) partly mediated mitochondrial fission leading to embryonic lethality at 11.5 dpc [50].

Above all, abnormal expression of SLC25A26 in oocytes and embryos can cause changes of SAM content in mitochondria, resulting in changed gene or protein methylation levels which could affect the expression of mitochondrial function gene like *mt-Cytb* and disturb mitochondria function, leading to reduced Δ*ψ*m, ATP content, and increased production of ROS, thereby affecting oocyte quality and embryonic development. However, oocyte maturation is inseparable from the periovicular environment *in vivo*. It is worth exploring whether abnormal expression of *Slc25a26* in oocyte has crosstalk with granulosa cell *in vivo*, and whether there is abnormal expression of *Slc25a26* in granulosa cells of aged female because impaired cellular functions in granulosa cells can also impact quality of oocyte and early embryo.

## 5. Conclusion

In summary, accumulation of SLC25A26 in mouse oocytes damaged oocyte quality through impairing mitochondrial function and mitochondrial respiratory chain. And deficiency of SLC25A26 in mice resulted in embryonic lethality at 10.5 dpc. Therefore, proper level of SLC25A26 in oocyte/embryo is essential for normal oocyte maturation and early embryo development.

## Figures and Tables

**Table 1:** Misregulated genes upon SLC25A26 overexpression in mouse oocytes.

Gene symbols	Chr	Description and function (source: UniProt)
*C1qc*	4	Complement C1q subcomponent subunit C. Complement activation, classical pathway source, and innate immune response [51].
*Ccl4*	11	Chemokine (C-C motif) ligand 4. Cytokine, chemotaxis, and inflammatory response [52].
*Ctss*	3	Cathepsin S. Antigen processing and presentation of exogenous peptide antigen via MHC class II Source [53].
*Tmsb4x*	X	Thymosin beta-4. Binds to and sequesters actin monomers (G actin) and therefore inhibits actin polymerization [54].
*Scimp*	11	SLP adaptor and CSK interacting membrane protein. Lipid tetraspanin-associated transmembrane adapter/mediator that acts as a scaffold for Src family kinases and other signaling proteins in immune cells [55].
*Pramel38*	5	PRAME-like 38. Negative regulation of apoptotic process, cell differentiation and transcription, and DNA-templated. Positive regulation of cell population proliferation. Source: InterPro.
*Mt-Cytb*	MT	Mitochondrially encoded cytochrome b. Component of the ubiquinol-cytochrome c reductase complex (complex III or cytochrome b-c1 complex) that is part of the mitochondrial respiratory chain [56].
*E330014E10Rik*	5	RIKEN cDNA E330014E10 gene. Negative regulation of apoptotic process, cell differentiation and transcription, and DNA-templated. Positive regulation of cell population proliferation. Source: InterPro.
*Pomc*	12	Proopiomelanocortin. A polypeptide hormone precursor yielding several biologically active peptides involved in different cellular functions.
*Prl*	13	Prolactin acts primarily on the mammary gland by promoting lactation.
*Nnat*	2	Neuronatin participates in brain development.
*Gh*	11	Growth hormone is involved in growth control.

## Data Availability

RNA-seq data generated during this study has been deposited to GEO database: PRJNA773040.

## Abbreviations

GV: Germinal vesicle
GVBD: Germinal vesicle breakdown
SAMC: S-adenosylmethionine carrier
SAM: S-adenosylmethionine
ROS: Reactive oxygen species
OXPHOS: Oxidative phosphorylation
SAH: S-adenosine homocysteine
HHcy: Hyperhomocysteinemia
ETC: Electron transport chain.

## Supplementary Materials

Supplementary Materials Supplementary Figure 1: Slc25a26 cRNA and H2b-egfp cRNA were successfully microinjected into oocytes. Representative images of EGFP fluorescence signals in oocytes 16 hours after microinjection with Slc25a26 cRNA and H2b-egfp cRNA. Scale bar = 50 *μ*m. Supplementary Figure 2: knockdown of Slc25a26 does not restore maturation rate of the aged oocytes. (A) Representative images of aged oocytes injected with control or siRNA against Slc25a26 (siSlc25a26). Scale bar = 50 *μ*m.(B) Relative mRNA levels of Slc25a26 were detected in control and siSlc25a26 group. (C) Quantitative analysis of GVBD and Pb1 extrusion rate in control and siSlc25a26 group. ^∗∗^*p* < 0.01. Data are presented as the mean ± SEM. Supplementary Figure 3: overexpression of SLC25A26 does not affect mitochondrial DNA copy number. Relative mtDNA copy numbers were detected in control and SLC25A26-OE oocytes. Supplementary Figure 4: PCA analysis of RNA-seq data from control and SLC25A26-OE oocytes at GV stage. Each dot represents one library, color-coded by oocyte group. Supplementary Figure 5: overexpression of SLC25A26 does not affect TE expression level. Comparison of normalized counts of TEs between control and SLC25A26-OE oocytes. Supplementary Figure 6: Slc25a26 cRNA and H2b-egfp cRNA were successfully microinjected into zygotes. Representative images of EGFP fluorescence signal in 4.5 dpc blastocyst microinjected with Slc25a26 cRNA and H2b-egfp cRNA. Scale bar = 50 *μ*m. Supplementary Figure 7: generation of the Slc25a26 KO allele using CRISPR/Cas9. Schematic representation of the mouse Slc25a26 locus with exon 2 deletion. Supplementary Figure 8: strategy of genotyping. Wild type: ② PCR reaction obtains a single WT band (440 bp). Heterozygote: ① PCR reaction obtains a KO band (246 bp) and ② PCR reaction obtains a WT band (440 bp). Homozygote: ① PCR reaction obtains a single KO band (246 bp) and ② PCR reaction without product.
