# Supplementary material for: Aberrant Expression of Mitochondrial SAM Transporter SLC25A26 Impairs Oocyte Maturation and Early Development in Mice
Source: Oxid Med Cell Longev. 2022 Apr 13;2022:1681623. doi: 10.1155/2022/1681623 (PMC9020962; doi:10.1155/2022/1681623)
Supplement: Supplementary Materials — Supplementary Figure 1: Slc25a26 cRNA and H2b-egfp cRNA were successfully microinjected into oocytes. Representative images of EGFP fluorescence signals in oocytes 16 hours after microinjection with Slc25a26 cRNA and H2b-egfp cRNA. Scale bar = 50 μm. Supplementary Figure 2: knockdown of Slc25a26 does not restore maturation rate of the aged oocytes. (A) Representative images of aged oocytes injected with control or siRNA against Slc25a26 (siSlc25a26). Scale bar = 50 μm.(B) Relative mRNA levels of Slc25a26 were detected in control and siSlc25a26 group. (C) Quantitative analysis of GVBD and Pb1 extrusion rate in control and siSlc25a26 group. ∗∗p < 0.01. Data are presented as the mean ± SEM. Supplementary Figure 3: overexpression of SLC25A26 does not affect mitochondrial DNA copy number. Relative mtDNA copy numbers were detected in control and SLC25A26-OE oocytes. Supplementary Figure 4: PCA analysis of RNA-seq data from control and SLC25A26-OE oocytes at GV stage. Each dot represents one library, color-coded by oocyte group. Supplementary Figure 5: overexpression of SLC25A26 does not affect TE expression level. Comparison of normalized counts of TEs between control and SLC25A26-OE oocytes. Supplementary Figure 6: Slc25a26 cRNA and H2b-egfp cRNA were successfully microinjected into zygotes. Representative images of EGFP fluorescence signal in 4.5 dpc blastocyst microinjected with Slc25a26 cRNA and H2b-egfp cRNA. Scale bar = 50 μm. Supplementary Figure 7: generation of the Slc25a26 KO allele using CRISPR/Cas9. Schematic representation of the mouse Slc25a26 locus with exon 2 deletion. Supplementary Figure 8: strategy of genotyping. Wild type: ② PCR reaction obtains a single WT band (440 bp). Heterozygote: ① PCR reaction obtains a KO band (246 bp) and ② PCR reaction obtains a WT band (440 bp). Homozygote: ① PCR reaction obtains a single KO band (246 bp) and ② PCR reaction without product. [file 1681623.f1.docx]

***Supplementary Material***

**Supplementary Figures**

**
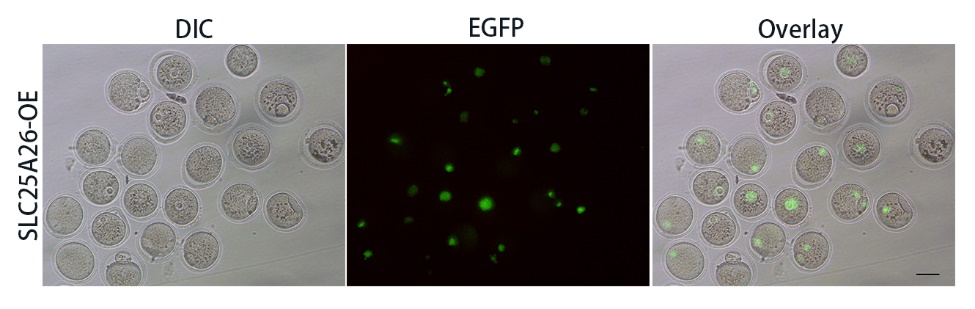
**

**Supplementary Figure 1. Slc25a26 cRNA and H2b-egfp cRNA were successfully microinjected into oocytes.** Representative images of EGFP fluorescence signals in oocytes 16 hours after microinjection with Slc25a26 cRNA and H2b-egfp cRNA. Scale bar=50 μm.

**
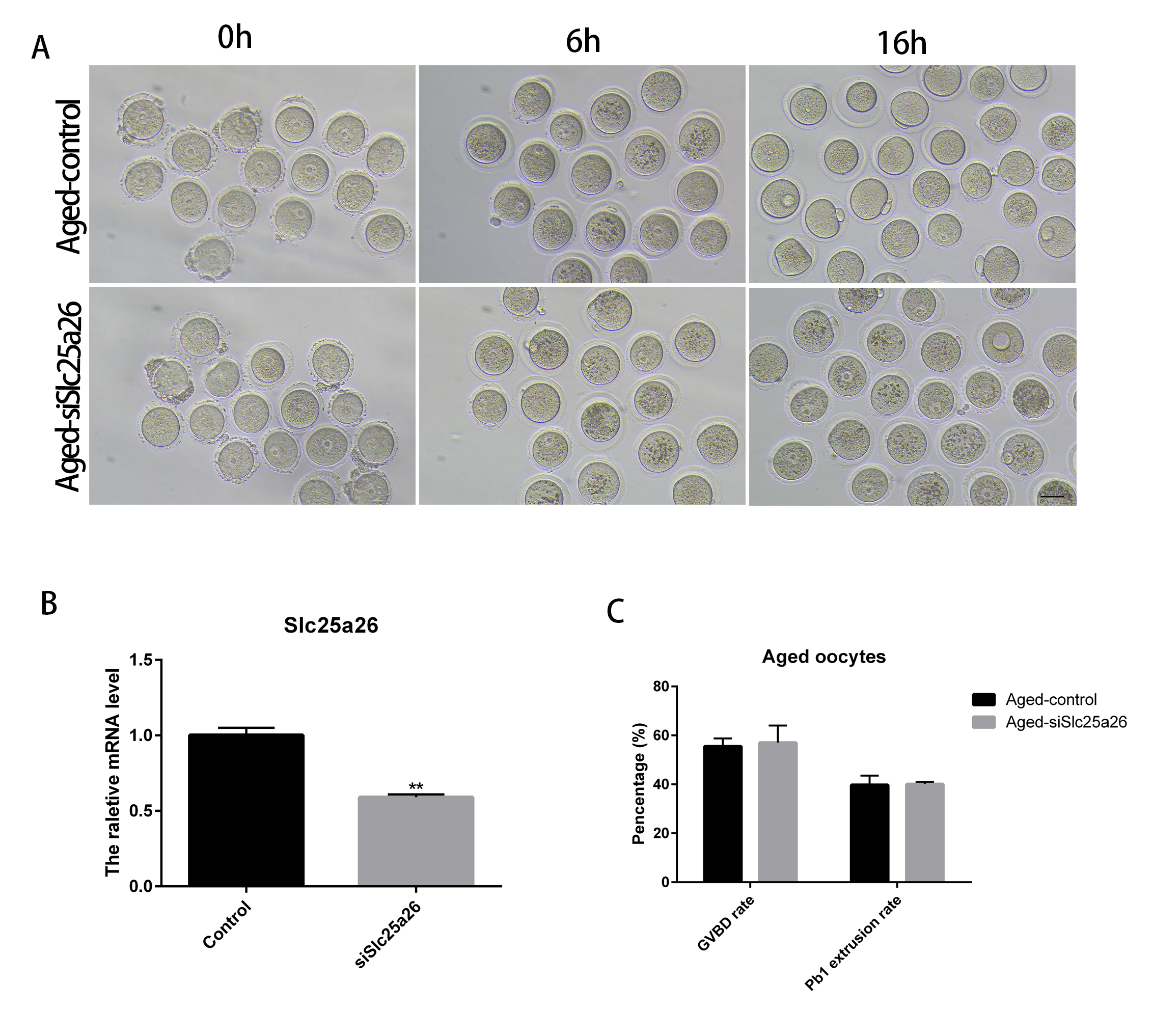
**

**Supplementary Figure 2. Knockdown of *Slc25a26* does not restore maturation rate of the aged oocytes.** (A) Representative images of aged oocytes injected with control or siRNA against *Slc25a26* (siSlc25a26). Scale bar=50 μm.(B) Relative mRNA levels of *Slc25a26* were detected in control and siSlc25a26 group. (C) Quantitative analysis of GVBD and Pb1 extrusion rate in control and siSlc25a26 group. **p<0.01. Data are presented as the mean ± SEM.





**Supplementary Figure 3.** **Overexpression of SLC25A26 does not affect mitochondrial DNA copy number.** Relative mtDNA copy numbers were detected in control and SLC25A26-OE oocytes.


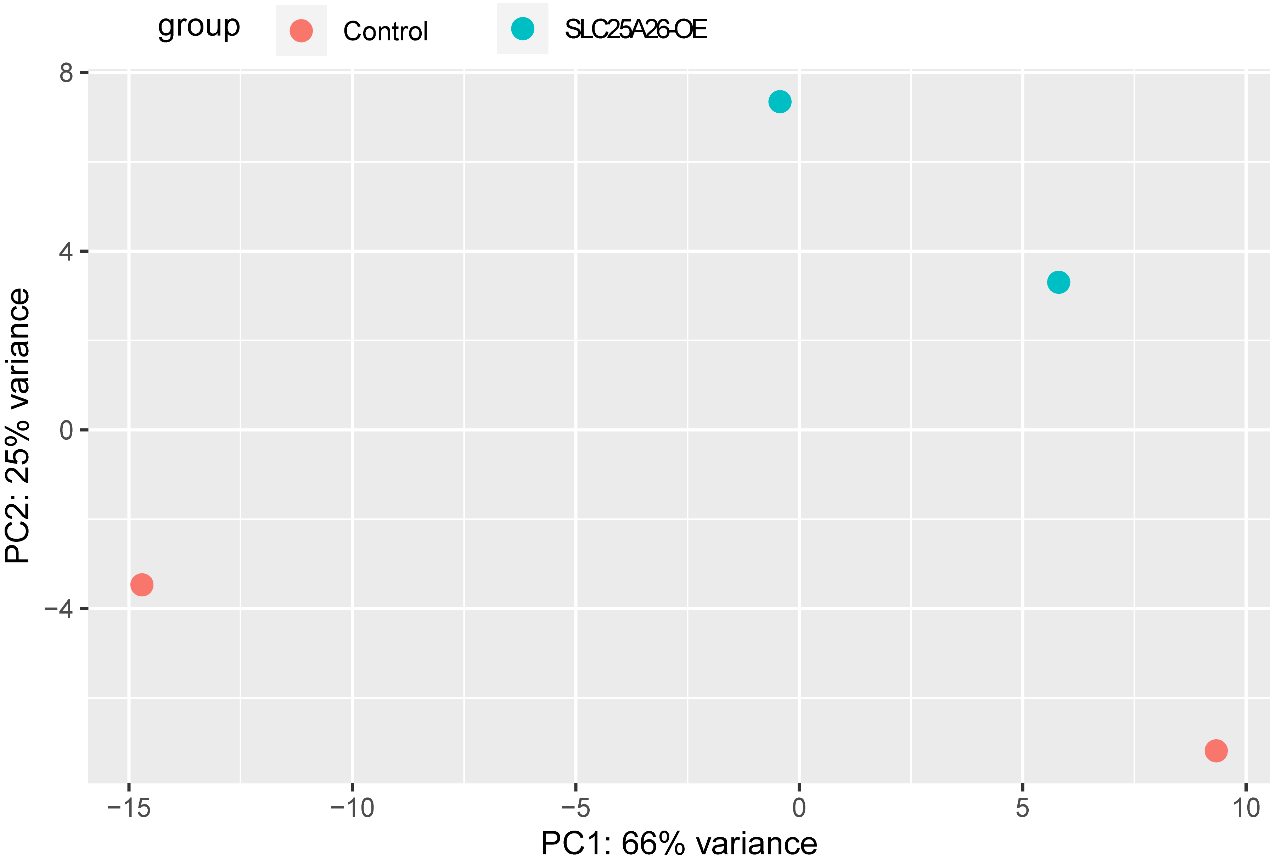


**Supplementary Figure 4.** PCA analysis of RNA-Seq data from control and SLC25A26-OE oocytes at GV stage. Each dot represents one library, color-coded by oocyte group.


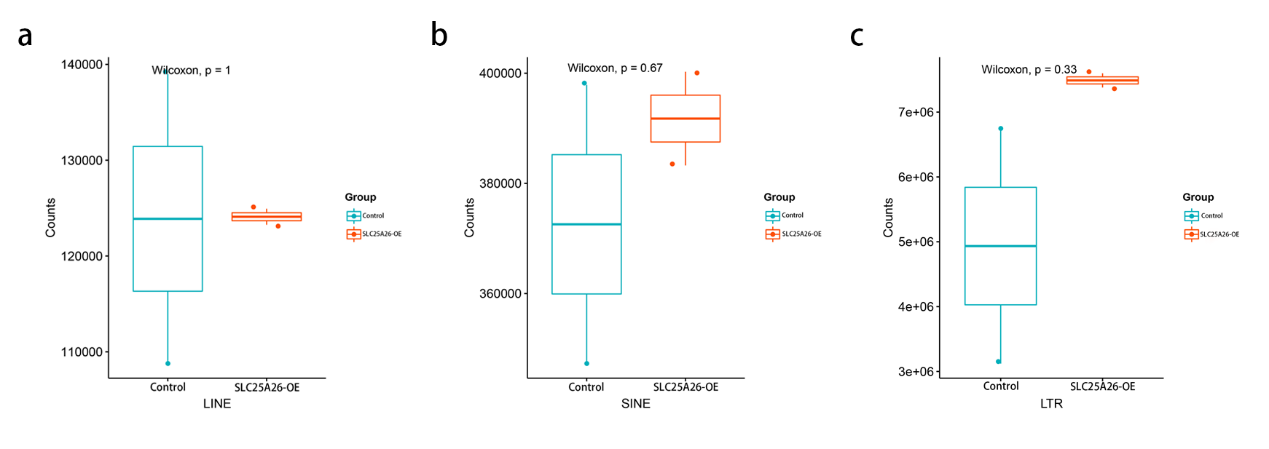


**Supplementary Figure 5.** **Overexpression of SLC25A26 does not affect TE expression level.** Comparison of normalized counts of TEs between control and SLC25A26-OE oocytes.


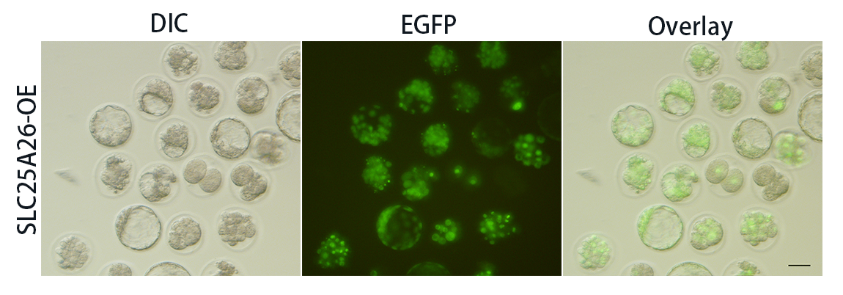


**Supplementary Figure 6. Slc25a26 cRNA and H2b-egfp cRNA were successfully microinjected into zygotes.** Representative images of EGFP fluorescence signal in 4.5dpc blastocyst microinjected with Slc25a26 cRNA and H2b-egfp cRNA. Scale bar=50 μm.


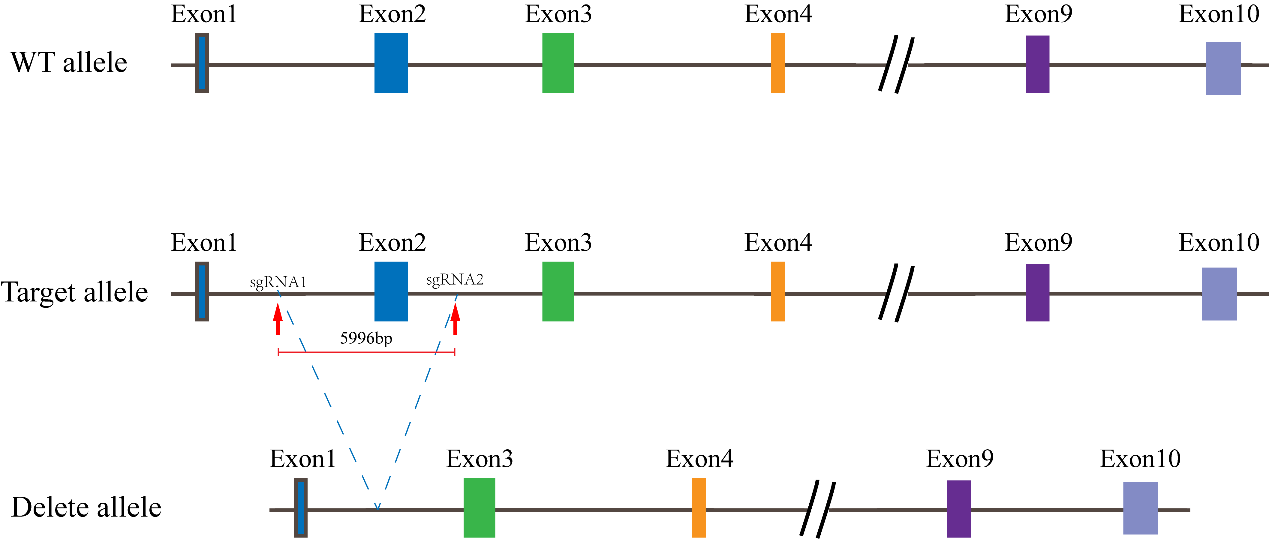


**Supplementary Figure 7. Generation of the *Slc25a26* KO allele using CRISPR/Cas9.** Schematic representation of the mouse *Slc25a26* locus with exon 2 deletion.


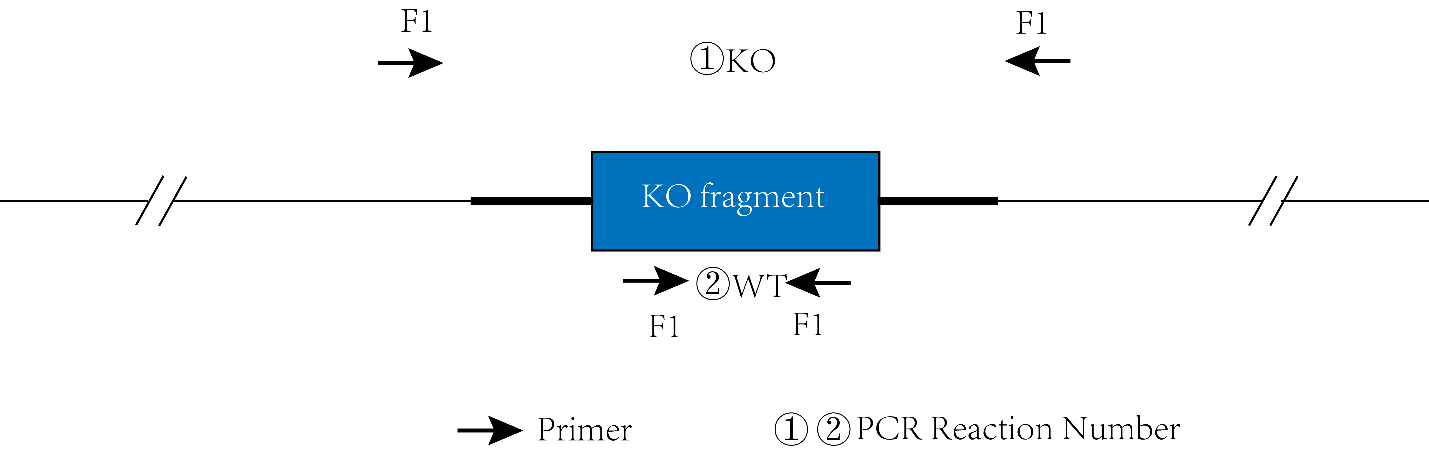


**Supplementary Figure 8. Strategy of Genotyping.**

Wild type: ②PCR reaction obtains a single WT band (440bp).

Heterozygote: ①PCR reaction obtains a KO band (246bp); ②PCR reaction obtains a WT band (440bp).

Homozygote: ①PCR reaction obtains a single KO band (246bp); ② PCR reaction without product.
